# The Case for Pulmonary Metastasectomy—Clinical Practice Narrative Review and Commentary

**DOI:** 10.3390/life14060702

**Published:** 2024-05-29

**Authors:** Paolo Scanagatta, Gianluca Ancona, Sara Cagnetti, Casimiro Eugenio Giorgetta, Francesco Inzirillo, Eugenio Ravalli, Martina Maiolani, Giuseppe Naldi

**Affiliations:** 1 Division of Thoracic Surgery, ASST Valtellina e Alto Lario, “Eugenio Morelli” Hospital, 23035 Sondalo, Italy; 2 Division of Oncology, ASST Valtellina e Alto Lario, “Ospedale Civile” Sondrio, 23100 Sondalo, Italy

**Keywords:** metastasectomy, pulmonary metastasectomy, lung metastases, lung cancer

## Abstract

Pulmonary metastasectomy has become a well-established procedure for patients with certain types of solid tumors. Patients are usually scheduled for staged lung metastasectomy in case of primary tumor control, the absence of distant non-lung metastases, and when complete resection is achievable. Nodules are removed with precision resection in order to ensure radical resection with minimal margins; this technique permits good oncological results, preserving the surrounding pulmonary parenchyma and causing minimal distortion compared to staplers. When possible, anatomical resections should be avoided since they are not justified by real oncological advantages and, in the majority of cases, sacrifice too much healthy tissue, possibly leading to inoperability in the case of metachronous relapses. Thus, preserving the maximum amount of pulmonary parenchyma is crucial because repeated metastasectomies are possible and frequent, with no theoretical limits to the number of reinterventions. In our multidisciplinary board team, we support the role of pulmonary metastasectomy as a useful curative therapy, with acceptable morbidity and mortality, with indications to be discussed case-by-case.

## 1. Introduction

The pulmonary parenchyma is a very common site of metastases, mainly due to anatomical and functional reasons, with high blood flow and a large surface area. The alveolar capillaries, slow-flow vessels, are the perfect site for free tumoral cells to implant. Metastases are expressions of a disseminated process, and the outcomes mainly depend on effective systemic therapy, but in selected cases, surgery may play a therapeutic role. One of the first reports of pulmonary metastasectomy dates back to 1882 [1]; since then, this procedure has been widely performed in oncologically advanced cases. In 1991, the International Registry of Lung Metastases reported an overall survival rate from 13% to 36%, depending on the primary histological nature [2]. Currently, pulmonary metastasectomy has become a standard procedure for pediatric patients with certain types of solid tumors [3], and it is considered a safe and potentially curative treatment with low morbidity and mortality [4].

The role of metastasectomy in adults is currently debated, and data have mainly been collected and analyzed for some kinds of solid tumors. Currently, it is consolidated for primary bone and soft tissue sarcomas; several studies have shown good results, increasing overall survival and disease-free survival [2,5,6,7,8].

The aim of this study is to analyze the data to better understand the current indications for metastasectomy of the lung in clinical practice according to the scientific literature and to give our interpretation.

## 2. Discussion

Pulmonary metastasectomy is a widely accepted therapeutic option in the management of lung metastases that, in strictly selected patients, can enhance the chance of long-term survival. This concept is mainly due to several studies that have investigated the benefits of repeated metastasectomy [9,10,11,12,13,14], and even the international work group of the European Society of Thoracic Surgeons (ESTS) reported similar results in a paper from 2010 [15].

Our experience and results suggest, as shown in other studies [16,17,18], that multiple staged bilateral and repeated metastasectomies are worth performing because of their possible favorable long-term survival. This is consistent with the concept of oligometastases. If the biology of cancerous cells does not change after the first metastasectomy, then we can assume that subsequent metastasectomies have similar efficacy. However, the international literature still debates the benefits of multiple subsequent metastasectomies.

In 2020, a paper by Yang et al. [9] reported a significantly higher rate of recurrence after the second re-metastasectomy. Likewise, Mineo et al. [19] described performing two to six subsequent metastasectomies for lung metastases, and in their impressions, the more resections, the greater the risk of recurrences and the shorter the disease-free time, possibly making repeated surgery less effective after the first procedure.

However, an improvement in the long-term survival of multiple subsequent metastasectomies has been described in several other studies. Cheung et al. [20] have described, in patients with sarcoma, an increased median survival after two or more repeat metastasectomies. Pastorino et al. in 2021 described how in osteosarcoma, the number of subsequent metastasectomies, if executed under rigorous criteria, does not act as a negative factor for OS [21]. Park et al. [22] have reported, in patients with colorectal cancer, a similar 5-year survival after the second and third metastasectomy. According to Matsumoto et al. (2019), after the first and second lung metastasectomy, there is no difference concerning the overall 5-year survival [10], whereas Hishida et al. [23] report a significant improvement in survival following the first repeated surgery compared to the second one.

The issue is further confounded by the different prognostic criteria taken into consideration in repeat metastasectomy from one study to another. Presumably, patients with a favorable outcome have not overrun a biological threshold of malignant disease. An interesting issue would be to identify this condition or biological or clinical boundaries not to be crossed. According to this, the identification of new biological markers or favorable/negative prognostic signs could be the target of future investigations.

**Figure 1:**
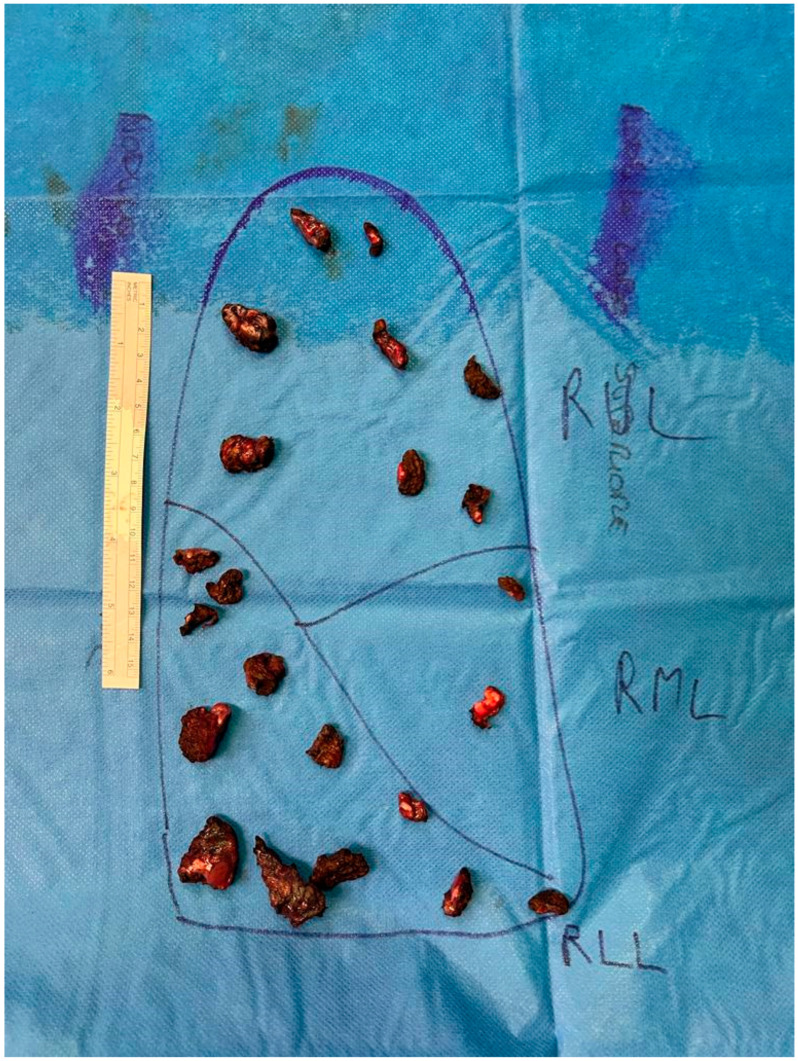
Intraoperative arrangement of 22 precision resections, containing 25 metastatic nodules. This image is provided to pathologists to assist them in correctly identifying and assessing the position of the different nodules.

Other publications on this issue are concordant, considering the number of metastases as a strong negative predictor [12,13,14,19], which is true. However, our experience shows that an increasing number of lesions just makes the identification of all the nodules more difficult, so manual palpation of all parenchyma is mandatory [24] (Figure 1).

Other authors have identified nodal status [11], the diameter of the largest metastasis [25], concomitant liver metastases [23], or disease-free survival [9,13,19] as negative predictors as well.

When approaching these data, it must be kept in mind that the vast majority of these studies have small numbers of patients or have a short follow-up. Definitive and official recommendations are limited due to a lack of randomized controlled trials. Randomized controlled trials with a double-blind approach and strictly regulated selection are an extremely powerful tool in the right hands, and the data they produce are strong evidence. However, we cannot always delay confirmation until the production of such complex and lengthy studies to confirm something we are confident in because of long-time clinical evidence. As a noticeable model, we would like to report the parachute example, for which there are also no confirmations due to the lack of enrollable subjects, probably due to selection bias, and no related studies are ongoing [26].

Another important piece of information to keep in mind is the selection bias among retrospective studies. Some of the major limitations include different adjuvant therapies, enrollment selections, and variations in follow-up length [27].

Recruitment bias, in our opinion, due to the particular psychological situation those patients are living in, cannot and will not be avoided in the future. Patients with metastatic malignancy frequently suffer from clinical or subclinical depression due to the lack of hope that transpires through medical doctors of all specialties. Often, they do not see any way out, and anyone who gives them even a small chance of survival is seen as a savior. Thus, the occurrence of patients refusing a surgical intervention that gives them the theoretical possibility of a longer and healthier life with their family is particularly rare. And what is this if not a selection bias? In fact, only one randomized controlled trial has been conducted and was closed early due to recruitment issues. Although the results cannot be statistically reliable due to incompleteness, the observed 5-year survival rate of 38% in metastasectomy patients compared to 29% in controls provides a compelling indication for more in-depth investigations [28].

The eligibility for pulmonary resection lacks strict criteria, but there are a few points all authors agree on [29]. First, the patient has to be a fit candidate for thoracic surgical intervention [2]. From an oncological perspective, the primary neoplasm must be under control or controllable [15], and there should be no extrathoracic metastases. However, if such metastases exist, they must also be completely resectable or have been successfully resected already [27]. Another important and not so obvious point is that surgery should aim to completely free the lung from all pulmonary metastases. Lastly, surgical intervention should be the best-proven therapeutic option available for treating the metastasis. In fact, with the fast evolution of targeted, immune, and other emerging therapies, this should be the most important consideration. These criteria are used mainly for the first surgical metastasectomy, considering both lungs if needed and with the minimum time in between. After surgery, the patient comes back to the oncologist to maintain the follow-up timing, usually with a thoracic CT scan after 3 months. As soon as new nodules appear on the CT scan, a new evaluation is needed. Oncologists have to evaluate if a new cycle of chemotherapy can be administered or if it is necessary to change drugs. The indications for a re-metastasectomy include the growth of already present nodules, because with the appearance of new nodules, the disease is not controlled by therapy and the oncological treatment needs to be adjusted before considering a local treatment. When and if the disease should become controlled after chemotherapy (at least with the stability of the nodule number at a chest CT scan), the patient can be evaluated for a new intervention. If surgery is not possible or is contraindicated for medical reasons, it should be evaluated by a multidisciplinary board and, if possible, the patient should be a candidate for stereotactic radiotherapy [30].

Another point of discussion is the surgical access and technique for a correct pulmonary metastasectomy. Regarding the access, many authors have documented the necessity for open surgery and the manual palpation of nodules in the lung. Marulli G. et al. stated that when palpation is not necessary, typically due to a limited number of easily identifiable lesions, the VATS approach is feasible even with precision techniques using laser or other energy devices [31]. One of the authors described this technique in a very interesting video on his personal channel (Droghetti A. n.d.r.). McCormack et al. discovered that bimanual palpation during VATS revealed additional lesions in half of patients [32], and other studies have shown that up to 46% of lesions palpated were not detected on preoperative CT scans [33,34] or even identified in the contralateral lung after unilateral disease was confirmed during preoperative staging [35]. In all of these studies, it is important to note that not all additional lesions are malignant. Furthermore, no study has yet proven a survival benefit from resecting lesions that CT scans failed to detect. Meanwhile, Kang et al. reported a significant enhancement in detecting subcentimeter nodules, achieving a sensitivity of 97% and a negative predictive value of 96% through the use of high-resolution CT scanning with 1 mm slices [36].

Recent studies have reported comparable survival and cancer recurrence rates between VATS and open techniques, which range from 31% to 69% [37,38]. These findings reveal no significant differences in survival, thus sparking a debate about the clinical relevance of resecting nodules that are undetectable radiologically. Regarding the technique, it is mandatory to consider the preservation of uninvolved pulmonary parenchyma, even considering the possibility of future reinterventions. This approach leads atypical resection (wedge) to negative margins to be the most common intervention. Employing staplers for multiple wedge resections results in distortion of the lobar architecture and leads to considerable loss of unaffected parenchyma. For this reason, fine-tip electrocautery resection, or even better, laser precision resection, are the alternatives that we commonly consider during open techniques. This is particularly useful when approaching patients with higher-burden (but still completely resectable) disease. The absence of mechanical sutures helps in the manual identification of small deposits, and the small parenchymal defects created are repaired by volumetric-tailored sutures, drastically reducing the distortion of lobar architectures [39,40]. Central or voluminous lesions may need a lobectomy, but the indications for extensive resection, however, require approval from a multidisciplinary board. Even the necessity of a pneumonectomy is not an absolute contraindication; however, it is crucial to consider that this procedure carries significant risks and is associated with variable long-term survival outcomes [41].

Another highly debatable point is the need for lymphadenectomy during pulmonary metastasectomy. While lymph node metastases can complicate pulmonary metastatic disease, quantifying the risk associated with N+ disease remains challenging. Additionally, the relationship between the number and size of pulmonary metastatic nodules and the risk of lymph node involvement has not been clearly established. Various papers have shown an incidence of unexpected LN involvement between 9.2% and 36% with high variance in different malignancy histologies [42,43]. LN involvement correlates with poorer survival outcomes, ranging from 0% to 24% over five years, while patients with negative LNs experience a 24.7% to 50% five-year survival rate [44]. The prognostic value of involvement at different LN stations remains unclear. A study on lung metastases from renal cancer indicated a median survival of approximately 64 months for N1 involvement, around 33 months for N2 disease, and 21 months for N3 involvement [45]. Other studies, however, have not demonstrated any differences in survival between the involvement of any lymph nodal stations [46].

Epithelial histologies seem to benefit more from lymphadenectomy, whereas such an advantage does not appear to be present in sarcomatous tumors (bone and soft tissue) [44,45,46].

The surgical approach is also not standardized: is it better to perform a systematic lymphadenectomy or lymph node sampling? Also, for primitive lung cancer, the study conducted by the American College of Surgery Oncology Group revealed no significant differences in outcomes between the two techniques [47].

The uncertain results of the scientific literature about the impact on prognosis of lymphadenectomy in metastatic patients are well reflected by the differences in approach observed in surgical practice. In the 1997 International Registry of Lung Metastases, lymph node metastases were reported in only 5% of patients, with complete systematic lymph nodal dissection conducted in only a minority of cases [2]. A more recent survey by the ESTS found that 55% of thoracic surgeons sampled lymph nodes, 13% performed a complete lymphadenectomy, and 3.2% did not examine any lymph nodal stations [48].

## 3. Conclusions

A standard-of-care approach for evaluating patients for pulmonary metastasectomy has not yet been established, underscoring the importance of a multidisciplinary review and case-by-case assessment.

Close interdisciplinary collaboration is essential for developing a therapy plan and monitoring oncological treatments. Systematic oncological follow-up, focused on the early detection and timely local treatment of recurrent metastases, may enhance survival in carefully selected patients.

Prospective studies and trials comparing surgical resection to systemic or targeted therapies and ablation will be crucial for progress in the field. As immunotherapy becomes increasingly common in treating locally advanced and metastatic cancers, its integration into standard treatment protocols for isolated pulmonary metastases and oligometastases will likely necessitate adjustments, potentially making surgery a default component.

VATS can be considered for metastasectomy of a limited number of nodules, while for a higher number of lesions, a small muscle-sparing thoracotomy may be preferred [49]. Could it lead to a new example of the Will Rogers phenomenon? It is not worth laughing about [50,51,52].

Many workgroups keep considering this aggressive approach with suspicion and are waiting for higher levels of evidence to introduce metastasectomy into their clinical practice. They are probably right, and researchers must keep analyzing results for a better understanding of this topic, but it is also true that the enrollment for parachute testers could be open soon … [26].

Any volunteers?

## References

[1] Weinlechner J.W. (1882). Tumoren an der brustwand und deren behnadlung resection der rippeneroffnung der brusthohle und partielle entfernung der lunge. Wien. Med. Wochenschr..

[2] Pastorino U., Buyse M., Friedel G., Ginsberg R.J., Girard P., Goldstraw P., Johnston M., McCormack P., Pass H., Putnam J.B. (1997). Long term results of lung metastasectomy: Prognostic analyses based on 5206 cases. J. Thorac. Cardiovasc. Surg..

[3] Temeck B.K., Wexler L.H., Steinberg S.M., McClure L.L., Horowitz M., Pass H.I. (1995). Metastasectomy for Sarcomatous Pediatric Histologies: Results and Prognostic Factors. Ann. Thorac. Surg..

[4] Kayton M.L. (2006). Pulmonary metastasectomy in pediatric patients. Thorac. Surg. Clin..

[5] Predina J.D., Puc M.M., Bergey M.R., Sonnad S.S., Kucharczuk J.C., Staddon A., Kaiser L.R., Shrager J.B. (2011). Improved survival after pulmonary metastasectomy for soft tissue sarcoma. J. Thorac. Oncol..

[6] Digesu C.S., Wiesel O., Vaporciyan A.A., Colson Y.L. (2016). Management of Sarcoma Metastases to the Lung. Surg. Oncol. Clin. N. Am..

[7] Shimizu J., Emori M., Murahashi Y., Sonoda T., Mishina T., Miyajima M., Watanabe A., Sugita S., Takada K., Murase K. (2020). Pulmonary metastasectomy is associated with prolonged survival among patients with bone and soft tissue sarcoma. Mol. Clin. Oncol..

[8] Gherzi L., Ferrari M., Pardolesi A. (2022). Lung metastases from sarcoma: Multidisciplinary approach—A narrative review. AME Surg. J..

[9] Yang K.M., Park I.J., Lee J.L., Kim C.W., Yoon Y.S., Lim S.-B., Yu C.S., Kim J.C. (2019). Benefits of repeated resections for liver and lung metastases from colorectal cancer. Asian J. Surg..

[10] Matsumoto T., Hasegawa S., Hida K., Kawada K., Sakai Y., Sugihara K. (2019). Role of repeat resection in patients with metastatic colorectal cancer: A multicenter retrospective study. Dis. Colon Rectum.

[11] Menna C., Berardi G., Tierno S.M., Andreetti C., Maurizi G., Ciccone A.M., D’Andrilli A., Cassiano F., Poggi C., Diso D. (2018). Do repeated operations for recurrent colorectal lung metastases result in improved survival?. Ann. Thorac. Surg..

[12] Salah S., Watanabe K., Park J.S., Addasi A., Park J.W., Zabaleta J., Ardissone F., Kim J., Riquet M., Nojiri K. (2013). Repeated resection of colorectal cancer pulmonary oligometastases: Pooled analysis and prognostic assessment. Ann. Surg. Oncol..

[13] Borasio P., Gisabella M., Billé A., Righi L., Longo M., Tampellini M., Ardissone F. (2011). Role of surgical resection in colorectal lung metastases: Analysis of 137 patients. Int. J. Color. Dis..

[14] Welter S., Jacobs J., Krbek T., Krebs B., Stamatis G. (2007). Long-term survival after repeated resection of pulmonary metastases from colorectal cancer. Ann. Thorac. Surg..

[15] Van Raemdonck D., Friedel G. (2010). The European Society of Thoracic Surgeons lung metastasectomy project. J. Thorac. Oncol..

[16] Sponholz S., Schirren M., Baldes N., Oguzhan S., Schirren J. (2017). Repeat resection for recurrent pulmonary metastasis of colorectal cancer. Langenbeck’s Arch. Surg..

[17] Hachimaru A., Maeda R., Suda T., Takagi Y. (2016). Repeat pulmonary resection for recurrent lung metastases from colorectal cancer: An analysis of prognostic factors. Interact. Cardiovasc. Thorac. Surg..

[18] Mizuguchi S., Nishiyama N., Izumi N., Tsukioka T., Komatsu H., Iwata T., Tanaka S., Takemura S., Kubo S. (2016). Clinical significance of multiple pulmonary metastasectomy for hepatocellular carcinoma. World J. Surg..

[19] Mineo T.C., Ambrogi V., Tacconi F., Mineo D. (2015). Multireoperations for lung metastases. Future Oncol..

[20] Cheung F., Alam N., Wright G. (2018). Pulmonary metastasectomy: Analysis of survival and prognostic factors in 243 patients. ANZ J. Surg..

[21] Pastorino U., Palmerini E., Porcu L., Luksch R., Scanagatta P., Meazza C., Leuzzi G., Massimino M., Picci P. (2023). Lung metastasectomy for osteosarcoma in children, adolescents, and young adults: Proof of permanent cure. Tumori J..

[22] Park J.S., Kim H.K., Choi Y.S., Kim K., Shim Y.M., Jo J., Lee W.-Y., Chun H.-K., Park Y.S., Kang W.K. (2010). Outcomes after repeated resection for recurrent pulmonary metastases from colorectal cancer. Ann. Oncol..

[23] Hishida T., Tsuboi M., Okumura T., Boku N., Ohde Y., Sakao Y., Yoshiya K., Hyodo I., Mori K., Kondo H. (2017). Does repeated lung resection provide longterm survival for recurrent pulmonary metastases of colorectal cancer? Results of a retrospective Japanese Multicenter Study. Ann. Thorac. Surg..

[24] Meazza C., Scanagatta P., Luksch R., Massimino M. (2015). How far can we go with surgery in metastatic osteosarcoma patients?. Med. Oncol..

[25] Yamamoto Y., Kanzaki R., Kanou T., Ose N., Funaki S., Shintani Y., Minami M., Outani H., Takenaka S., Hamada K. (2019). Long-term outcomes and prognostic factors of pulmonary metastasectomy for osteosarcoma and soft tissue sarcoma. Int. J. Clin. Oncol..

[26] Smith G., Pell J. (2003). Parachute use to prevent death and major trauma related to gravitational challenge: Systematic review of randomised controlled trials. BMJ.

[27] Handy J.R., Bremner R.M., Crocenzi T.S., Detterbeck F.C., Fernando H.C., Fidias P.M., Firestone S., Johnstone C.A., Lanuti M., Litle V.R. (2019). Expert consensus document on pulmonary metastasectomy. Ann. Thorac. Surg..

[28] Treasure T., Farewell V., Macbeth F., Monson K., Williams N.R., Brew-Graves C., Lees B., Grigg O., Fallowfield L. (2019). Pulmonary Metastasectomy versus Continued Active Monitoring in Colorectal Cancer. Trials.

[29] Kondo H., Okumura T., Ohde Y., Nakagawa K. (2005). Surgical treatment for metastatic malignancies. Pulmunary metastasis: Indications and outcomes. Int. J. Clin. Oncol..

[30] Meazza C., Scanagatta P. (2016). Metastatic osteosarcoma: A challenging multidisciplinary treatment. Expert Rev. Anticancer Ther..

[31] Marulli G., Droghetti A., Di Chiara F., Calabrese F., Rebusso A., Perissinotto E., Muriana G., Rea F. (2013). A prospective randomized trial comparing stapler and laser techniques for interlobar fissure completion during pulmonary lobectomy. Lasers Med. Sci..

[32] McCormack P.M., Bains M.S., Begg C.B., Burt M.E., Downey R.J., Panicek D.M., Rusch V.W., Zakowski M., Ginsberg R.J. (1999). Role of video-assistedthoracic surgery in the treatment of pulmonary metastases: Results of a prospective trial. Ann. Thorac. Surg..

[33] Kayton M.L., Huvos A.G., Casher J., Abramson S.J., Rosen N.S., Wexler L.H., Meyers P., LaQuaglia M.P. (2006). Computed tomographic scan of the chest underestimates the number of metastatic lesions in osteosarcoma. J. Pediatr. Surg..

[34] Cerfolio R.J., McCarty T., Bryant A.S. (2009). Non-imaged pulmonary nodules discovered during thoracotomy for metastasectomy by lung palpation. Eur. J. Cardiothorac. Surg..

[35] Long H., Zheng Y., Situ D., Ma G., Lin Z., Wang J. (2011). Hand-assisted thoracoscopic surgery for bilateral lung metastasectomy through sternocostal triangle access. Ann. Thorac. Surg..

[36] Kang M.C., Kang C.H., Lee H.J., Goo J.M., Kim Y.T., Kim J.H. (2008). Accuracy of 16-channel multi-detector row chest computed tomography with thin sections in the detection of metastatic pulmonary nodules. Eur. J. Cardiothorac. Surg..

[37] Carballo M., Maish M.S., Jaroszewski D.E., Holmes C.E. (2009). Video-assisted thoracic surgery (VATS) as a safe alternative for the resection of pulmonary metastases: A retrospective cohort study. J. Cardiothorac. Surg..

[38] Gossot D., Radu C., Girard P., Le Cesne A., Bonvalot S., Boudaya M.S., Validire P., Magdeleinat P. (2009). Resection of pulmonary metastases from sarcoma: Can some patients benefit from a less invasive approach?. Ann. Thorac. Surg..

[39] Pereszlenyi A., Rolle A., Koch R., Schilling A., Baier B., Bis B. (2005). Resection of multiple lung metastases—Where are the limits?. Bratisl. Lek. Listy.

[40] Scanagatta P., Girelli L. (2017). Metastasectomy in pediatric patients: Indications, technical tips and outcomes. J. Thorac. Dis..

[41] Spaggiari L., Grunenwald D.H., Girard P., Solli P., Le Chevalier T. (1998). Pneumonectomy for lung metastases: Indications, risks, and outcome. Ann. Thorac. Surg..

[42] Hamaji M., Cassivi S.D., Shen K.R., Allen M.S., Nichols F.C., Deschamps C., Wigle D.A. (2012). Is lymph node dissection required in pulmonary metastasectomy for colorectal adenocarcinoma?. Ann. Thorac. Surg..

[43] Seebacher G., Decker S., Fischer J.R., Held M., Schäfers H.-J., Graeter T.P. (2015). Unexpected lymph node disease in resections for pulmonary metastases. Ann. Thorac. Surg..

[44] Reinersman J.M., Wigle D.A. (2016). Lymphadenectomy during Pulmonary Metastasectomy. Thorac. Surg. Clin..

[45] Pfannschmidt J., Hoffmann H., Muley T., Krysa S., Trainer C., Dienemann H. (2002). Prognostic factors for survival after pulmonary resection of metastatic renal cell carcinoma. Ann. Thorac. Surg..

[46] Renaud S., Falcoz P.E., Alifano M., Olland A., Magdeleinat P., Pagès O., Regnard J.F., Massard G. (2014). Systematic lymph node dissection in lung metastasectomy of renal cell carcinoma: An 18 years of experience. J. Surg. Oncol..

[47] Darling G.E., Allen M.S., Decker P.A., Ballman K., Malthaner R.A., Inculet R.I., Jones D.R., McKenna R.J., Landreneau R.J., Rusch V.W. (2011). Randomized trial of mediastinal lymph node sampling versus complete lymphadenectomy during pulmonary resection in the patient with N0 or N1 (less than hilar) non-small cell carcinoma: Results of the American College of Surgery Oncology Group Z0030 Trial. J. Thorac. Cardiovasc. Surg..

[48] Internullo E., Cassivi S.D., Van Raemdonck D., Friedel G., Treasure T. (2008). Pulmonary metastasectomy: A survey of current practice amongst members of the European Society of Thoracic Surgeons. J. Thorac. Oncol..

[49] Durkovic S., Scanagatta P. (2015). Muscle-Sparing Thoracotomy: A Systematic Literature Review and the “Ave” Classification. J. Surg. Surg. Res..

[50] Feinstein A.R., Sosin D.M., Wells C.K. (1985). The Will Rogers phenomenon: Stage migration and new diagnostic techniques as a source of misleading statistics for survival in cancer. N. Engl. J. Med..

[51] Sormani M.P. (2009). The Will Rogers phenomenon: The effect of different diagnostic criteria. J. Neurol. Sci..

[52] Stander M., Stander J. (2020). A simple method for correcting for the Will Rogers phenomenon with biometrical applications. Biom. J..

